# Supplementary material for: Patterns of asymmetry and energy cost generated from predictive simulations of hemiparetic gait
Source: PLoS Comput Biol. 2022 Sep 9;18(9):e1010466. doi: 10.1371/journal.pcbi.1010466 (PMC9491609; doi:10.1371/journal.pcbi.1010466)
Supplement: Attachment — Submitted filename: review_final.pdf [file pcbi.1010466.s002.pdf]

This is a simulation study that seeks to determine whether asymmetric gait post-stroke is in part an energetically optimal adaptation to unilateral muscle weakness. They use a focused 'lesion' in-silico approach that creates unilateral muscle weakness in the lower extremities, akin to reduced neural drive seen in stroke. They then measure the effect of degrees of muscle weakness on metabolic cost and gait symmetry across a range of speeds. In accordance with their expectations, muscle weakness leads to optimal gait solutions that are asymmetric. If one were to enforce symmetry, despite the muscle weakness, metabolic cost increases. Taken together, the asymmetry may be an optimal choice given the weakness. However, the asymmetry does not explain the increased metabolic cost observed in stroke.

Generally, such movement simulation studies can be very informative despite the many assumptions that must be made. Specifically, optimization studies like this one are particularly useful as they allow us to probe the underlying cost function. Thus I believe this study, even with its limited focus and assumptions about the nature of stroke impairments (see below), can be quite valuable for stroke as well as other impairments. The main critique is that the baseline simulation data for a healthy individual does not match well with decades of experimental data. Without strong confidence that they can replicate healthy walking, it is hard to accept the simulation results for 'stroke' walking. Additional modeling analyses are needed to better validate their models and strengthen their conclusions.

General Comments:

1. The baseline data shown in Figure 2 do not match well with experimental data measures in healthy humans walking at these speeds.
   1a. For example, the vertical ground reaction forces in the 1.0 and 1.25 m/s conditions have peaks of over 2 BW. This is very high. The shape is also more asymmetric than that observed previously for these relatively standard gait speeds. It also isn't clear why the vertical GRF does not clearly drop to 0. This may be a figure editing issue with the thickness of the lines, but it doesn't seem so.
   1b. Secondly, for the horizontal GRF the curves are also quite different than expected. Zooming in on the y-axis may help clarify things, but without zooming in again it seems like the curves are quite asymmetric. Shouldn't the braking and push-off be symmetric? Otherwise how is the model walking at a constant speed? There is also a concern here for why the forces do not clearly drop to 0 in the latter half of the gait cycle. Does the model not have a single-stance phase? Perhaps a movie of their simulation would help convince readers that this gait is normal.
   1c. COT is also quite high. I don't see support for a COT of 10 J/(kg.m) in the citations noted(line 309). 10 J/(kg.m) seems quite high, even though it is such a slow speed. Even at 0.5 m/s the COT is quite high given that there is no upper body. For reference, Koelewijn et al. (PLoS One 2019) show a COT of 1.05 (Umberger) for 0.8m/s and 1.11 for 1.3m/s.
2. There are a number of assumptions in the modeling approach which the researchers adequately address throughout the article, however, these assumptions do limit the impact of their finding. By using a cost function that minimizes effort cost and fits to

asymmetries, the solutions produced will be impacted by the heuristically-determined weighting of these factors. The article could use additional analysis to strengthen their results.

2a. Namely, there should be a sensitivity analysis of the cost weights used to fit the gait. Does varying the relative weights impact the resulting gait patterns and conclusions?

2b. Secondly, there should be an assessment of the quality of each fit and solutions found to verify the solutions are all sound.

2c. Finally, the article could be strengthened by additional discussion and analysis comparing model-produced gait patterns, asymmetries, and metabolic differences to those measured in stroke patients.

3. Putting aside the accuracy of the baseline model, and looking at the main results, I would like the authors to speak more to the relevance of their findings. It would help to map the degree of weakness they are simulating to what is observed in the actual stroke population.

   3a. For example, it seems that there really isn't any asymmetry until 40% or 60% muscle weakness. It would be helpful if the authors would put this in context. How prevalent is this degree of weakness in an individual that can still actually walk? Is it possible to see 40%-60% muscle weakness in someone who can walk, albeit asymmetrically? Putting these results in context would help clarify the significance of these findings.

   3b. More generally, there should be some justification for all the weakness levels tests and for how this maps onto functionality in the stroke population.

   3c. There should also be some justification for the weakness across all muscles and not just certain ones, but this is a minor point.
4. I may be mis-reading, but my understanding is that the optimal solution found my minimizing muscle activations cubed is representation of a metabolically optimal solution. That is, that this optimal solution should also be the solution that has the lowest COT. If this is indeed the case, then the authors should confirm that indeed, out of all the simulation for a given condition (weakness/speed) the solution that minimized muscle activations cubed also minimized COT.
5. General comment about the premise: is this a fair model of stroke? Are stroke patients still capable of optimizing and generating the optimal movement they determine or are they limited in other respects such as neural control, spasticity, and co-activation? I realize the authors do address this possibility/limitation in the introduction, but I feel that throughout the MS this is often forgotten and strong links are made between these results and implications for stroke. My comment here is mainly to simply say that they should be more aware of this limitation and temper their statements.

Specific Comments

1. Is a 2-D model fair given the extent of the asymmetry? I would imagine that such high levels of weakness would induce a large amount of movement in the frontal plane. But it is difficult to assess without a good grasp on the severity of this 60% weakness on gait.

2. A table with all the simulation conditions would be helpful. It should list the weaknesses and the speeds, as well as what is tested in the forced symmetry. The forced symmetry conditions tested were especially confusing.
3. Line 214: ‘but NOT step time asymmetry’ rather than ‘but step time asymmetry’
4. Figure 2 and Figure 3 have the colors in the legend reversed.
5. Lines 303-304: Are these straight numerical comparisons? I don’t see how the 1.00 m/s is the opposite. It would be helpful to report the numbers being compared since the figures tend to be zoomed out or have lines overlapping so it is difficult to compare.
6. In Figure 4, how was the COT for each limb calculated? I am confused why the COT in figure 4A is not the sum of the COT in figures 4B and 4C.
7. 187: “Theoretically” may be incorrectly used here. Perhaps “ideally” is a more appropriate term?
8. 184, 199, 350, throughout: There is some inconsistency with using the terms step length/time symmetry/asymmetry throughout the paper. This is also exacerbated by using the acronyms SLA and STA, then referring to them as “step length symmetry”. Additionally, “reduce step time symmetry” is used in a few locations (184, 305), which is counterintuitive and possibly a typo, especially given the preceding statement of “reducing these asymmetries is a common…” (180, 186).
9. 215: Why were model fits only performed at 0.75 m/s? Why was that chosen? How would results at 1.5 m/s differ?
10. 295: Consider doing a quality of fit assessment to explain away this outlier fit at 40%/1.25m/s
11. Fig5. The rationale for not including a data point at 1.5 m/s for 40% and 60% weakness makes sense, but using that logic, a fit should be included for 1.5 m/s and 20% weakness, because it does not have a clear minimum similar to the symmetric.
12. 350: Is this a typo? “Minimizing step length symmetry”. Shouldn’t this be minimizing asymmetry?
13. Fig 4A-C, Fig5, Fig 6C: What is the uncertainty in the metabolic model? Are these points statistically different (especially Fig 6C)?
