# Supplementary material for: Patterns of asymmetry and energy cost generated from predictive simulations of hemiparetic gait
Source: PLoS Comput Biol. 2022 Sep 9;18(9):e1010466. doi: 10.1371/journal.pcbi.1010466 (PMC9491609; doi:10.1371/journal.pcbi.1010466)
Supplement: Attachment — Submitted filename: PLOS Review Response.docx [file pcbi.1010466.s003.docx]

**Simulated hemiparesis increases optimal spatiotemporal gait asymmetry but not metabolic cost**

**Reviewer 1:**

In this study, the investigators used 2D musculoskeletal modeling to investigate relationships between muscle strength, metabolic cost, and spatiotemporal gait symmetry. The results suggest that asymmetric walking patterns may be energetically optimal for persons with unilateral lower extremity weakness. This manuscript will be of interest to the biomechanics community and the readership of PCB. My comments and suggestions are included below:

ABSTRACT

In the abstract and throughout the manuscript, I recommend focusing on unilateral weakness rather than stroke. After reading the first sentence of the abstract, I was surprised that this manuscript used a model developed based on musculoskeletal parameters of healthy gait. While unilateral weakness is clearly relevant to stroke, I find the conclusions drawn about stroke gait to be a bit speculative given that many other common post-stroke deficits (e.g., spasticity, muscle atrophy, muscle stiffness, changes in tendon morphology, etc) are not addressed in the modeling and could significantly affect interpretations of this line of work. To this reviewer, it seems more appropriate to focus on the impact of unilateral weakness on interactions between cost and asymmetry while relegating potential applications to stroke to the discussion.

This comment is also relevant to the title, given that conditions other than paresis (e.g., fatigue) could a reduced ability to generate muscle forces unilaterally. I suggest revising to “Simulated unilateral weakness increases…”

1.1) Thank you for this comment on clarifying the scope of our study. We have reframed the manuscript to focus on hemiparesis, defined as unilateral muscle weakness, rather than focusing on stroke. We modified both our abstract and introduction to highlight other clinical populations where hemiparesis is relevant (see changes **Lines 41-80**).

INTRODUCTION

Related to my comment above, I recommend that the authors reframe the introduction around unilateral weakness rather than stroke.

1.2) As noted in comment 1.1 and based on your suggestion, we have reframed the introduction to center around hemiparesis as a common impairment that occurs in a range of different clinical populations. (**Lines 41-80**)

It would be helpful if the authors could provide some context in the introduction about why weakness in particular – of the many common unilateral gait deficits – was chosen. I also think it is important to provide some background on prior studies of muscle weakness and their relationships to the parameters of interest here (e.g., asymmetry, energy cost). As currently written, the topic of weakness is not addressed at any length until the final paragraph of the introduction.

1.3) We've identified weakness as an important component seen in clinical populations, in part, because previous work has tried to understand the correlations between muscle strength and gait deviations. While these studies have provided value to the literature, it can be hard to interpret the findings due to other confounding neuromuscular deficits which are also present in different populations. Additionally, weakness is a common target of rehabilitation interventions and is often associated with gait asymmetries. Therefore, we need to better understand the isolated effects of unilateral weakness on gait performance.

**Lines 59 - 65** - "Previous researchers have tried to identify how specific neuromuscular impairments relate to measures of gait performance by using regression or correlation analyses. Hemiparesis in the knee extensors and hip flexors after a stroke has been correlated with reductions in gait speed and increases in step length asymmetries (25–28). Similarly, spatiotemporal asymmetries have been shown to correlate positively with the energy cost of walking post-stroke (15). Despite the abundance of work examining relationships between impairment and measures of gait performance post-stroke, one key limitation of these approaches is that regression analyses are insufficient to determine the causal effects of any specific impairment."

**Lines 71 – 73** - "Additionally, improving muscle strength is a common target for rehabilitation protocols as a way to improve spatiotemporal asymmetries, however it’s still unclear if improvements in muscle strength lead to improvements in gait performance (36,37)."

Expansion on prior findings where musculoskeletal modeling has led to insight into the effect of impairments on gait mechanics and energetics (line 72) would be helpful.

1.4) Recent work has, for example, shown that weak plantar flexor muscles are directly related to walking with a "heel walking" gait. We have referenced this work in the introduction as follows.

**Lines 79 - 81** - "For example, these studies have helped identify that weak plantar flexor muscle groups can explain calcaneal or “heel-walking” gait patterns, as seen in children with spastic diplegia (36,41)."

METHODS

Why was the DeGroote model chosen over the many other available musculoskeletal models?

1.5) To solve optimal control problems, Moco uses gradient-based optimization which requires models that are smooth and continuous everywhere. The DeGroote muscle model was used in this study since it is designed to be efficient with gradient-based optimization (i.e, smooth and continuous muscle curves).

**Lines 110-115**: "The *DeGrooteFregly2016Muscle* model was used for this study since it is smooth and continuous everywhere; this is required for Moco which uses a gradient-based optimization approach to solve optimal control problems. In addition, it provides an implicit form of muscle-tendon dynamics, which is more computationally efficient and converges more reliably compared to the explicit form of muscle-tendon dynamics (53)."

The choice of a 2D model makes extrapolation of the findings to neurologic populations (where there are commonly out-of-plane compensatory movements) difficult. What was the justification for selecting a 2D model? This is justified somewhat in the limitations section of the discussion, but I advise caution in justifying such choices based on what was best for the modeling/software (e.g., time cost of simulations) vs. what was best for answering the research question. This also applies to the justification for the investigation of weakness in the discussion (“…because hemiparesis is straightforward to model”).

1.6) We were primarily interested in understanding the relationships between weakness and step time/step length asymmetry. Since these asymmetries result from deviations in the sagittal plane, restricting the model to move in the sagittal plane allowed us to reduce the number of confounding variables in our analysis. Besides the computational advantages of working only with as complex of a model as required for our primary variables (i.e., using a 2-D model to understand sagittal plane deviations), a 3-D analysis will require additional tests to understand the validity and potential weaknesses of a more complex musculoskeletal model. Additional research is necessary to begin to separate sagittal plane deviations from other types of gait deviations such as hip hiking or circumduction.

**Line 489 - 491**: "Since these asymmetries result from deviations in the sagittal plane, restricting the model to move in the sagittal plane only allowed us to reduce the number of confounding variables in our analysis."

What was the rationale for systematically reducing all muscles by the same relative magnitude? Is there scientific justification for this in clinical work?

1.7) We chose to reduce all muscles by the same relative magnitude for two primary reasons: 1. As our first foray into this space, we made a decision to create uniform weakness conditions to keep our analyses simple as there is limited published data on patterns of muscle-level weakness in hemiparetic populations. 2. One potential limitation of changing strength by joint/degree of freedom is it's difficult to assess how to weaken muscles that act across multiple joints. Future work could look at how different combinations of weakness patterns affect gait patterns. We added our explanation for this rationale in the text as follows.

**Line 125 - 129**: "Finally, we chose to reduce all muscles on the limb by the same magnitude within each condition for two reasons: 1. It allows us to keep our analysis relatively simple with mild, moderate, and more severe levels of weakness across the three hemiparetic models, and 2. A limitation of changing strength per joint is that it can be difficult to assess how to weaken muscles that act across multiple joints."

**Line 539 - 542:** "While we modeled a uniform level of muscle weakness within each hemiparetic model (i.e., all muscles on the left limb were reduced by the same magnitude) future work could study how different combinations of muscle weakness affect gait performance"

Given that co-contraction and spasticity are common in post-stroke gait (and other neurologic conditions with unilateral weakness), is it an appropriate optimization technique to minimize muscle excitations?

1.8) Thank you for this point. We minimized muscle excitations with the assumption that effort is an important factor for producing voluntary movements in humans. Co-activation of muscles could be considered a constraint that emerges due to reliance on descending control pathways that couple muscles within and across joints (e.g., see Dewald et al., 1995 and Sanchez et al., 2018). However, it is conceivable that reducing effort remains an important objective in post-stroke gait even in the presence of this obligatory co-activation. While people post-stroke do exhibit other impairments, this study focused on a single impairment (weakness) to develop the specific correlations between the variables: weakness, asymmetry, and energy. While it is not within the scope of our study, better understanding the role of co-activation of muscles in clinical populations is a future direction of this line of research, especially with respect to recent evidence that co-activation may be effort-optimal in systems with uncertainty (Koelewijn and van den Bogert 2022).

**Line 156 - 158**: "We chose to minimize muscle excitations cubed based on the hypothesis that effort is an important factor for producing voluntary movements in humans, including individuals with neuromuscular impairments."

**Lines 500 - 507**: "Additionally, co-activation of muscles and muscle spasticity, which often presents together with hemiparesis, can also affect the gait performance in individuals with neuromuscular impairments. Co-activation of muscles could be considered a constraint that emerges due to reliance on descending control pathways that couple muscles within and across joints (e.g., 13,14). However, it is conceivable that reducing effort remains an important objective in post-stroke gait even in the presence of this obligatory co-activation. While people with hemiparesis do exhibit other impairments, this study focused on a single impairment (unilateral weakness) to develop the specific correlations between the variables: weakness, asymmetry, and metabolic energy."

RESULTS

I am curious about the relationships between the simulated muscle activity and the spatiotemporal asymmetries, in particular the nonmonotonic relationships between step time asymmetry, weakness, and speed. It seems as though there must be conditions where weakness in certain muscle groups (e.g., plantarflexors) must drive the spatiotemporal and kinematic gait changes to a larger degree than others. Have the authors considered investigating any of these types of relationships?

1.9) As briefly mentioned in comment 1.7, we focused on uniform levels of muscle weakness in the left limb because it allowed us to have interpretable levels of impairment across conditions (e.g., mild, moderate, severe). However, other predictive simulation work has focused on specific muscle groups in isolation, for example Falisse et al., (2019) reduced the strength of the hip muscles bilaterally which resulted in a Trendelenburg type of gait pattern (characterized by excessive frontal plane rotation of the pelvis segment). Both Falisse et al., and Ong et al., (2019) have studied the relationship between bilaterally weak plantar flexor muscles and gait patterns, and both found that they result in a "calcaneal" gait pattern (characterized by increased ankle dorsiflexion which is maintained throughout stance). Future studies can explore how different patterns of hemiparesis across joints results in particular gait deviations, given that the effects of impairments at a single joint may not sum linearly.

**Line 539 - 542:** "While we modeled a uniform level of muscle weakness within each hemiparetic model (i.e., all muscles on the left limb were reduced by the same magnitude) future work could study how different combinations of muscle weakness affect gait performance"

The interactions between speed and cost are similarly interesting given their nonmonotonic trends. Is there any explanation for these relationships? It seems unusual and unexpected that the cost decreased with weakness when walking at 1.0 m/s (if I am interpreting lines 303-305 correctly).

1.10) Overall, we had a U-shaped relationship for the metabolic cost of transport for each model which is expected from experimental data on the relationship between gait speed and metabolic cost of transport. While there were some subtle differences within a speed across the models, the values are so close to one another that we interpret them overall as being practically equivalent.

Are there any GRF results to report? Given the relevance of the AP GRFs in neurologic populations, it would be interesting to see these data if available. These data would also be a nice complement to the mechanical work data.

1.11) We've plotted the AP and vertical GRFs for the symmetrical conditions across speeds in figures 2D and 2E. The other GRF data sets are available at our project webpage.

DISCUSSION

The discussion is comprehensive and well-written. I do not have any revisions to suggest beyond those that may be needed in addressing the comments above.

1.12) Thank you!

**Reviewer 2:**

This is a simulation study that seeks to determine whether asymmetric gait post-stroke is in

part an energetically optimal adaptation to unilateral muscle weakness. They use a focused

‘lesion’ in-silico approach that creates unilateral muscle weakness in the lower extremities, akin

to reduced neural drive seen in stroke. They then measure the effect of degrees of muscle

weakness on metabolic cost and gait symmetry across a range of speeds. In accordance with

their expectations, muscle weakness leads to optimal gait solutions that are asymmetric. If one

were to enforce symmetry, despite the muscle weakness, metabolic cost increases. Taken

together, the asymmetry may be an optimal choice given the weakness. However, the

asymmetry does not explain the increased metabolic cost observed in stroke.

Generally, such movement simulation studies can be very informative despite the many

assumptions that must be made. Specifically, optimization studies like this one are particularly

useful as they allow us to probe the underlying cost function. Thus I believe this study, even

with its limited focus and assumptions about the nature of stroke impairments (see below), can

be quite valuable for stroke as well as other impairments. The main critique is that the baseline

simulation data for a healthy individual does not match well with decades of experimental data.

Without strong confidence that they can replicate healthy walking, it is hard to accept the

simulation results for ‘stroke’ walking. Additional modeling analyses are needed to better

validate their models and strengthen their conclusions.

General Comments:

1. The baseline data shown in Figure 2 do not match well with experimental data measures

in healthy humans walking at these speeds.

1a. For example, the vertical ground reaction forces in the 1.0 and 1.25 m/s conditions

have peaks of over 2 BW. This is very high. The shape is also more asymmetric than that

observed previously for these relatively standard gait speeds. It also isn’t clear why the

vertical GRF does not clearly drop to 0. This may be a figure editing issue with the

thickness of the lines, but it doesn’t seem so.

2.1a) These peaks are typical in predictive simulations in which the objective function is purely effort-based, and generally occur because the model doesn't care about slapping its foot on the ground (no pain receptors). There are some ways to address this, but they have different disadvantages (e.g. a limit on max GRF would simply produce whatever maximum a user sets) and require an important, but subjective modeling decision on our part. Therefore, we have elected to keep these results as they are while acknowledging the discrepancy with observed experimental data in the discussion.

**Lines 510-521:** "Overall, the kinematics and ground reaction forces for our results match well with experimental gait patterns for neurotypical adults (Fig. 2), however there were a couple deviations from experimental data observed in our results. During mid-stance, the model has a nearly fully-extended knee (~0°; Fig 1B), while typical human gait patterns involve some mild knee flexion during mid-stance (~10 - 20°; (71)). It is possible that using an objective function that weights muscle excitations times muscle volume (rather than only muscle excitations) could induce more knee flexion during stance (e.g., (40,84)). The other kinematic deviation that occurs is at the hip angle, where the model never achieves hip hyperextension during gait, as is typically seen in human subjects. This is likely due to a modeling choice to have the pelvis and trunk locked together (e.g., no lumber flexion angle) to reduce the total number of degrees-of-freedom in the model and increase the computational speed for each optimization. The consequence of this choice was that both the lumbar and pelvis were tilted forward throughout the stride (Fig. 3), which resulted in an increase in the hip angle relative to the femur."

Regarding the lingering GRFs for the slower speeds. The optimal results for speeds of 0.25 and 0.50 m/s are to have a long stance phase and a short swing phase. It basically leaves the model's toe on the ground for a longer period of time to conserve effort for these conditions.

In addition, we've added experimental data to our Figure 2 to help readers understand where the similarities and differences lie for our baseline data. The black lines and gray shaded regions show reference experimental data of control human participants walking at 1.1 m/s. We can roughly compare these data with our predictive simulation results to qualitatively identify the similarities and differences between our results and experimental results.

1b. Secondly, for the horizontal GRF the curves are also quite different than expected.

Zooming in on the y-axis may help clarify things, but without zooming in again it seems

like the curves are quite asymmetric. Shouldn’t the braking and push-off be symmetric?

Otherwise how is the model walking at a constant speed? There is also a concern here

for why the forces do not clearly drop to 0 in the latter half of the gait cycle. Does the

model not have a single-stance phase? Perhaps a movie of their simulation would help

convince readers that this gait is normal.

2.1b) Thanks for your comment. We've changed the y-axis in the horizontal GRF figure so that it's easier to read. The results for the slower speeds (0.25 and 0.50 m/s) do have quite a short swing phase, as these are very slow gait speeds, the optimal gait pattern is to have the foot on the ground for a longer period of time. The faster speeds (e.g., 1.25 m/s) have a similar swing phase to our experimental reference data shown now in this figure.

While we do enforce the average speed across the gait cycle, the model is not walking at a constant speed throughout the stride. The gait speed will vary throughout the stride, but will average to our enforced speed. The fluctuation of gait speed within a stride is typical of steady state walking.

We've added a new figure (Figure 3) to our manuscript of snapshots of the model walking in the baseline condition at 1.00 m/s. We believe this is a useful addition to the manuscript to show the model walking normally. For readers who would be interested in seeing these results in more detail, all of our results are available at our project webpage for download.

1c. COT is also quite high. I don’t see support for a COT of 10 J/(kg.m) in the citations

noted(line 309). 10 J/(kg.m) seems quite high, even though it is such a slow speed. Even

at 0.5 m/s the COT is quite high given that there is no upper body. For reference,

Koelewijn et al. (PLoS One 2019) show a COT of 1.05 (Umberger) for 0.8m/s and 1.11 for

1.3m/s.

2.1c) Thank you for raising this concern about the metabolic COT. We've compared our results with some other references. For example Finley et al., 2017 shows a range of metabolic cost between 2 and 10 J/kg/m across a wide range of speeds for healthy control individuals when walking at speeds matched to post-stroke individuals (Fig 1), so we believe our results match up well with this range of experimental data. For modeling studies, Ong et al., 2019 showed a COT of ~4.5 J/kg/m at 0.5 m/s which is close to our results and matches with the experimental data well. The 0.25 m/s speed is quite slow, so a greater COT is expected. The Koelewijn et al (2019) paper, has a metabolic cost between 2 to 3 J/kg/m for walking speeds of 0.8 and 1.1 m/s, which does match with our data well. The values of 1.05 and 1.11 are root-mean-squared errors from Table 1 in that paper, but are not in units of J/kg/m.

2. There are a number of assumptions in the modeling approach which the researchers

adequately address throughout the article, however, these assumptions do limit the

impact of their finding. By using a cost function that minimizes effort cost and fits to

asymmetries, the solutions produced will be impacted by the heuristically-determined

weighting of these factors. The article could use additional analysis to strengthen their

Results.

2.2) In this study, the goal of our second analysis was to understand if attempts to minimize step time or step length asymmetry for an average speed would lead to increased metabolic cost. In developing this part of our project, we observed that this two-term objective function (muscle effort and symmetry) was the best way to approach the optimal control problem. We also tried using a constraint on the spatiotemporal symmetry terms, but this failed to have good convergence. We heuristically chose weightings for the two objective function terms based on how well the results minimized the spatiotemporal asymmetry and the quality of the convergence metrics.

**Line 239 - 241:** "These weightings were chosen heuristically such that we were able to reduce the asymmetry in each domain while getting good convergence. We noticed that if either w1 or w2 were too large, the optimization would not converge in a reasonable amount of time."

2a. Namely, there should be a sensitivity analysis of the cost weights used to fit the gait.

Does varying the relative weights impact the resulting gait patterns and conclusions?

2.2a) As referenced in comment 2.2, we chose weightings for the effort and spatiotemporal symmetry that resulted in a meaningful reduction in asymmetry and also gave us a converged solution in a reasonable amount of time. A lower weighting on the symmetry term resulted in gait patterns that were not adequately symmetrical, while significantly greater weightings on the symmetry term would typically not converge well.

2b. Secondly, there should be an assessment of the quality of each fit and solutions

found to verify the solutions are all sound.

2.2b) We've added experimental data to our Figure 2 which shows how well the baseline conditions align with expected control participants. For the second, spatiotemporal analysis, we evaluated the goodness of the result based on how well the optimal control result reduced the spatiotemporal asymmetries. We should also clarify that these simulations are fully predictive and do not fit to any experimental data.

2c. Finally, the article could be strengthened by additional discussion and analysis

comparing model-produced gait patterns, asymmetries, and metabolic differences to

those measured in stroke patients.

2.2c) Thanks for this comment. We have the following passages in our discussion section 4.2. Clinical Implications:

**Line 441-448:** "We know that spatiotemporal asymmetries, such as step length asymmetry, in people post-stroke are heterogeneous, with some individuals having longer step lengths with their paretic limb than their non-paretic and others having shorter step lengths with their paretic limb than non-paretic limb (35,78), however what drives these responses remains unclear (77). Our results suggest that one of the driving factors of asymmetry could be the level of hemiparesis, which could be tested via an experimental study by measuring the relationship between muscle strength asymmetries and spatiotemporal asymmetries during gait at different speeds."

**Line 453 - 464**: "However, it’s still an open question whether this goal could be considered the optimal way to walk for people post-stroke, with recent data suggesting that symmetrical step lengths do not improve measures of metabolic COT (33,35) and result in other kinematic and kinetic asymmetries (32). The results of our predictive simulations are consistent with these studies: when we enforced step length symmetry for the 0.75 m/s speed, the resulting gait pattern had significant step time asymmetry, with a small increase in metabolic COT. The experimental results in combination with our simulated results are reasonable given the underlying anatomical system is asymmetric, therefore optimal gait patterns are also likely to be asymmetric. However, that the symmetry conditions resulted in only small increases in metabolic COT suggest that there could be multiple gait strategies for people post-stroke that result in similar consumption of metabolic energy. Therefore, patients who prioritize a symmetrical appearance of gait may be able to perform their preferred gait pattern without a large penalty on their endurance."

**Line 476 - 483**: "Our data suggest that moderate speeds of 0.50 and 0.75 m/s result in effort-optimal gait patterns with moderate levels of gait asymmetry (<10% asymmetry indices), while slower (0.25 m/s) or faster speeds (1.00 or 1.25 m/s) can result in levels of gait asymmetry greater than 10%. Therefore, we can hypothesize that people post-stroke may walk with slower gait speeds since this allows them to maintain lower gait asymmetry while also keeping metabolic COT low. Further explanation of the preferred gait patterns in people post-stroke could be explained by factors like balance or comfort, though how people post-stroke sense and perceive these factors is an important question to be addressed but is beyond the scope of this study."

3. Putting aside the accuracy of the baseline model, and looking at the main results, I

would like the authors to speak more to the relevance of their findings. It would help to

map the degree of weakness they are simulating to what is observed in the actual stroke

population.

3a. For example, it seems that there really isn’t any asymmetry until 40% or 60% muscle

weakness. It would be helpful if the authors would put this in context. How prevalent is

this degree of weakness in an individual that can still actually walk? Is it possible to see

40%-60% muscle weakness in someone who can walk, albeit asymmetrically? Putting

these results in context would help clarify the significance of these findings.

2.3a) According to Sanchez et al., 2011 NNR, ambulatory post-stroke patients have muscle weakness asymmetries of as much as 60% between limbs, especially seen in the knee and ankle joints. Therefore, our conditions do represent the levels of muscle weakness asymmetry seen in post-stroke patients who are able to walk.

**Line 129-132:** "We chose the level of muscle weakness (i.e., 20 to 60% unilateral weakness) based on muscle torque data from Sanchez et al., 2017 (14), that showed that ambulatory post-stroke patients can have inter-limb muscle strength asymmetries of as much as 60%."

3b. More generally, there should be some justification for all the weakness levels tests

and for how this maps onto functionality in the stroke population.

2.3b) We chose the range of 20 to 60% muscle weakness for our models based on inter-limb torque data from people post-stroke from Sanchez et al., 2017 NNR. The data from this study shows ambulatory post-stroke patients can have sagittal plane weakness of up to 60% in the knee and ankle joints.

**Line 129-132:** "We chose the level of muscle weakness (i.e., 20 to 60% unilateral weakness) based on muscle torque data from Sanchez et al., 2017 (14), that showed that ambulatory post-stroke patients can have inter-limb muscle strength asymmetries of as much as 60%."

3c. There should also be some justification for the weakness across all muscles and not

just certain ones, but this is a minor point.

2.3c) As with comment 1.9: we chose to reduce the strength by a uniform number for simplicity in our analysis, and difficulty with deciding on muscles that cross multiple joints.

**Line 125 - 129: "**Finally, we chose to reduce all muscles on the limb by the same magnitude within each condition for two reasons: 1. It allows us to keep our analysis relatively simple with mild, moderate, and more severe levels of weakness across the three hemiparetic models, and 2. A limitation of changing strength per joint is that it can be difficult to assess how to weaken muscles that act across multiple joints."

**Line 539 - 542:** "While we modeled a uniform level of muscle weakness within each hemiparetic model (i.e., all muscles on the left limb were reduced by the same magnitude) future work could study how different combinations of muscle weakness affect gait performance"

4. I may be mis-reading, but my understanding is that the optimal solution found my

minimizing muscle activations cubed is representation of a metabolically optimal

solution. That is, that this optimal solution should also be the solution that has the

lowest COT. If this is indeed the case, then the authors should confirm that indeed, out

of all the simulation for a given condition (weakness/speed) the solution that minimized

muscle activations cubed also minimized COT.

2.4) While the optimal solution obtained from minimizing muscle excitations may be different from the optimal solution for metabolic cost minimization, we believe that our choice of cost function is appropriate for several reasons. Minimizing muscle excitations (or activations) is a common surrogate for metabolic cost in musculoskeletal simulations as it is strongly related to metabolic cost and performs well in gradient-based optimization. Minimizing muscle activation has been shown to produce realistic kinematics, muscle activity patterns, and cost of transport values in simulations of running (Miller et al., 2012). Furthermore, predictive simulations of walking that minimize metabolic cost can also benefit from minimizing muscle activations to produce realistic muscle activity (Falisse et al., 2019). Therefore, we believe that minimizing muscle excitations is a reasonable objective for the purposes of our study. For each walking condition in our study, we post-processed the solution to find the computed metabolic COT produced by the simulation, which is a common approach used by other simulation studies (Uchida et al. 2016, Dembia et al. 2017).

5. General comment about the premise: is this a fair model of stroke? Are stroke patients

still capable of optimizing and generating the optimal movement they determine or are

they limited in other respects such as neural control, spasticity, and co-activation? I

realize the authors do address this possibility/limitation in the introduction, but I feel

that throughout the MS this is often forgotten and strong links are made between these

results and implications for stroke. My comment here is mainly to simply say that they

should be more aware of this limitation and temper their statements.

2.5) As noted in responses 1.1 and 1.2, we have reframed our study to be modulating hemiparesis specifically, which is a common neuromuscular impairment that occurs in people post-stroke as well as a range of other clinical conditions. The language in the introduction and discussion has been updated to reflect this change.

Additionally, we've added some language to the discussion about other types of impairments and how that could play a role in changing gait patterns.

**Lines 500 - 507**: "Additionally, co-activation of muscles and muscle spasticity, which often presents together with hemiparesis, can also affect the gait performance in individuals with neuromuscular impairments. Co-activation of muscles could be considered a constraint that emerges due to reliance on descending control pathways that couple muscles within and across joints (e.g., 13,14). However, it is conceivable that reducing effort remains an important objective in post-stroke gait even in the presence of this obligatory co-activation. While people with hemiparesis do exhibit other impairments, this study focused on a single impairment (unilateral weakness) to develop the specific correlations between the variables: weakness, asymmetry, and metabolic energy."

Specific Comments

1. Is a 2-D model fair given the extent of the asymmetry? I would imagine that such high

levels of weakness would induce a large amount of movement in the frontal plane. But

it is difficult to assess without a good grasp on the severity of this 60% weakness on gait.

2.2.1) Noted from response 1.6 - We were primarily interested in understanding the relationships between weakness and step time/step length asymmetry. Since these asymmetries result from deviations in the sagittal plane, restricting the model to move in the sagittal plane allowed us to reduce the number of confounding variables in our analysis. Additional research would be needed to start separating other types of gait deviations such as hip hiking or circumduction from spatiotemporal asymmetries.

**Line 489 - 491**: "Since these asymmetries result from deviations in the sagittal plane, restricting the model to move in the sagittal plane only allowed us to reduce the number of confounding variables in our analysis."

2. A table with all the simulation conditions would be helpful. It should list the weaknesses

and the speeds, as well as what is tested in the forced symmetry. The forced symmetry

conditions tested were especially confusing.

2.2.2) We've added a table to Figure 1 to show the conditions we solved for across our study.

3. Line 214: ‘but NOT step time asymmetry’ rather than ‘but step time asymmetry’

2.2.3) Thank you, this is fixed.

4. Figure 2 and Figure 3 have the colors in the legend reversed.

2.2.4) Thank you. The legend has been fixed and made consistent for figures 2, 4 and 5 (used to be Figures 2 - 4).

5. Lines 303-304: Are these straight numerical comparisons? I don’t see how the 1.00 m/s

is the opposite. It would be helpful to report the numbers being compared since the

figures tend to be zoomed out or have lines overlapping so it is difficult to compare.

2.2.5) Thank you, we agree that this statement was confusing. We have updated it to clarify.

**Line 323 - 327:** "The metabolic COT was greater at 1.25 m/s than 1.00 in the 40% (3.57 J/kg/m at 1.00 vs 4.66 J/kg/m at 1.25 m/s) and 60% weakness (2.93 J/kg/m at 1.00 vs 3.55 J/kg/m at 1.25 m/s) models, while the metabolic COT was greater at 1.00 m/s than 1.25 in the symmetrical (3.55 J/kg/m at 1.00 vs 3.22 J/kg/m at 1.25 m/s) and 20% weakness models (3.56 J/kg/m at 1.00 vs 3.26 J/kg/m at 1.25 m/s)."

6. In Figure 4, how was the COT for each limb calculated? I am confused why the COT in

figure 4A is not the sum of the COT in figures 4B and 4C.

2.2.6) Thank you for this question. With our metabolic models, we are able to compute the metabolic cost for each individual muscle, as well as the basal metabolic rate. The COT for each limb is computed by summing the metabolic cost across each of the muscles in the respective limbs. The total metabolic cost in our model is composed of the sum of the metabolic cost of transport for the right limb, the metabolic COT for the left limb, plus the basal metabolic rate. For simplicity, we haven't shown the basal metabolic rate, but it is inversely proportional to the stride time for the optimal result. We've clarified this in the figure caption for Figure 5 (previously Figure 4).

Figure 5 Caption: "Note that the total metabolic COT (A) is the sum of the metabolic COT of the right limb (B), left limb (C), and the basal metabolic rate (not shown)."

7. 187: “Theoretically” may be incorrectly used here. Perhaps “ideally” is a more

appropriate term?

2.2.7) "Theoretically" changed to "ideally" [Line 206]

8. 184, 199, 350, throughout: There is some inconsistency with using the terms step

length/time symmetry/asymmetry throughout the paper. This is also exacerbated by

using the acronyms SLA and STA, then referring to them as “step length symmetry”.

Additionally, “reduce step time symmetry” is used in a few locations (184, 305), which is

counterintuitive and possibly a typo, especially given the preceding statement of

“reducing these asymmetries is a common…” (180, 186).

2.2.8) Thanks for finding these inconsistencies. We've gone through the document and updated our language to be more consistent.

**Line 204:** "allowed us to reduce step time asymmetry or step length asymmetry"

Equation 6: Abbreviations changed to SLS (Step Length Symmetry) and STS (Step Time Symmetry).

**Line 372:** "Minimizing step length asymmetry…"

9. 215: Why were model fits only performed at 0.75 m/s? Why was that chosen? How

would results at 1.5 m/s differ?

2.2.9) We chose to perform the second analysis at 0.75 m/s for a couple of reasons. First, 0.75 is close to the average walking speed for people post-stroke reported in recent studies (e.g., Sanchez and Finley, 2018). Secondly, the 0.75 m/s results in the nominal conditions allowed us to very systematically manipulate both the step length and step time symmetry results, since the nominal conditions had close to a linear relationship between muscle weakness level and step length asymmetry.

**Line 230 - 233:** "We chose to run this symmetry sub-analysis on the 0.75 m/s speed because the nominal results had a consistent pattern of increasing step length asymmetry with greater hemiparesis. We also chose this speed for this analysis because it aligns closely with the average gait speed reported in recent studies for patients post-stroke (35)."

10. 295: Consider doing a quality of fit assessment to explain away this outlier fit at

40%/1.25m/s

2.2.10) Thank you for this comment. We have further evaluated this result by running additional optimizations with different initial guesses for this condition (40% weakness at 1.25 m/s). We did additional optimizations with other initial guesses because one possible explanation is that the solution we have here is a local, but not global optimum. However, we found that this result was robust to multiple initial guesses, therefore we believe the result for this condition is correct to the best of our modeling assumptions.

11. Fig5. The rationale for not including a data point at 1.5 m/s for 40% and 60% weakness

makes sense, but using that logic, a fit should be included for 1.5 m/s and 20%

weakness, because it does not have a clear minimum similar to the symmetric.

2.2.11) Thank you for this idea. However, after considering it, we chose not to present the 20% weakness condition as we were unable to get consistent convergence at this speed with this level of weakness. While the level of weakness for the 20% model is mild, it did not reliably converge to a consistent solution, therefore we'd prefer not to present this result. We are still confident that the 1.25 m/s condition represents a minimum for this level of muscle weakness.

12. 350: Is this a typo? “Minimizing step length symmetry”. Shouldn’t this be minimizing

Asymmetry?

2.2.12) Yes, it was a typo. This is now fixed.

13. Fig 4A-C, Fig5, Fig 6C: What is the uncertainty in the metabolic model? Are these points

statistically different (especially Fig 6C)?

2.2.13) There is some uncertainty in the metabolic models that we used, as researchers have developed these models using mathematical representations of physiological processes. The original manuscripts for the Umberger metabolic probe (Umberger 2003 and 2010) present the testing and validation of this model, along with other recent literature that evaluates different types of metabolic models (e.g., Koelewijn et al., 2017 PLOS ONE). However, this topic is beyond the scope of this paper. With our predictive simulations resulting in single data point per condition, our typical statistical tests do not work well for assessing differences. Instead, we have provided context throughout the discussion on the relative differences between conditions, and related them to the magnitude of differences that would be meaningful for clinical populations.
